# Effects of exogenous 2,4-epibrassinolide on photosynthetic traits of 53 cowpea varieties under NaCl stress

**DOI:** 10.1515/biol-2022-0906

**Published:** 2024-10-24

**Authors:** Zhihui Hu, Xiaoping Liang, Zuyun Gong, Yanjie Wang, Chunxing Wu

**Affiliations:** College of Life Sciences, Jianghan University, Hubei Province Engineering Research Center for Legume Plants, Wuhan, Hubei 430056, China

**Keywords:** cowpea, 2,4-epibrassinolide, NaCl stress, photosynthetic traits, difference study

## Abstract

This study examined the effects of exogenous 2,4-epibrassinolide (EBR) on photosynthetic traits of 53 cowpea varieties under NaCl stress. The results of different analysis and correlation analysis showed that these 53 germplasm resources had rich genetic diversity, and significant correlations existed among various photosynthetic traits. Under NaCl stress, Pn was highly significantly positively correlated with Gs and Tr and extremely significantly negatively correlated with Ci. Under EBR treatment, Pn was extremely significantly positively correlated with Gs, Ci, Tr and it was significantly negatively correlated with Chla, Chlb, Chl(a + b), and Y(II). Under EBR treatment and NaCl stress, Pn was extremely significantly positively correlated with Tr, and significantly positively correlated with Gs and carotenoid reflectance index. Principal component analysis shows that in CK group and EBR treatment group, cowpea photosynthesis traits can be summarized as six principal components, contributing 82.298 and 83.046%, respectively, can replace 19 photosynthetic traits to evaluate 53 cowpea varieties; under NaCl stress group and EBR + NaCl stress group, photosynthesis traits can be summarized as seven principal components, with cumulative contribution rate of 84.564 and 85.742%, respectively. In the untreated case, the cluster analysis was used to screen 32 cowpea varieties exhibiting the strongest photosynthetic capacity. Under salt stress, six of these varieties were classified as salt-tolerant. Under EBR spraying + salt stress, all four varieties showed strong photosynthetic capacity, and EBR showed the best relief of salt stress. The results of this study will provide a theoretical basis for the application of exogenous EBR to alleviate cowpea salt stress damage.

## 1 Introduction

Cowpea (*Vigna unguiculata* Linn.) is vigna species of cowpea, papilionoid of legume. There are about 150 species of Vigna plants distributed in tropical regions [1]. Under short-term salt stress conditions, cowpea itself will form a defense mechanism in response to waterlogging, drought, and salt stress, and produce enzymatic and non-enzymatic antioxidant defense systems (antioxidant enzymes, antioxidants, and others) and osmoprotective substances (such as soluble sugar and soluble proteins) to alleviate the oxidative damage caused by salt stress [2,3]. However, with the prolongation of salt stress, cells will accumulate a large amount of reactive oxygen species (ROS) and break ROS metabolic system balance, thus aggravating membrane lipid peroxidation and inactivating biological macromolecules such as proteins and enzymes in cells, further resulting in the damage to the structure and function of cell membranes, the decreased photosynthetic capacity, and the hindered carbohydrate synthesis, eventually affecting the morphogenesis of crop reproductive development [4,5].

The natural plant hormone 2,4-epibrassinolide (EBR) is involved in plant cell division and elongation, photomorphogenesis, flowering, and the formation of yield and quality [6]. A large number of studies have shown that EBR can effectively remove excess ROS, prevent excessive oxidation of cells, and effectively alleviate abiotic stress damage caused by adversity stress in crops. EBR can improve the electron transfer rate of optical system and assign photosynthetic energy to photochemical reaction by improving the antioxidant system of plants [7,8]. However, there are few reports on the effects of exogenous EBR on physiological indicators of cowpea under salt stress. In this study, using 53 cowpea varieties as materials, a hydroponic experiment was carried out to investigate the effects of EBR pretreatment on physiological parameters such as photosynthetic parameters (net photosynthetic rate [Pn], intercellular CO_2_ concentration [Ci], stomatal conductance [Gs], and transpiration rate [Tr]), photosynthetic pigments (chlorophyll a [Chla], chlorophyll b [Chlb], and carotenoids [Car]), spectral parameters (actual photosynthetic efficiency [Y(II)], photochemical quenching [qP], non-photochemical quenching [NPQ], and variable/maximal fluorescence intensity [Fv/Fm]), fluorescence parameters (anthocyanin reflectance index [ARI1], carotenoid reflectance index [CRI1], photochemical reflectance index [PRI], and vegetation senescence reflectance index [PSRI]), and photosynthetic enzyme (ribulose 1,5-biphosphate carboxylase [Rubisco]) of cowpea seedlings under salt stress. This study also examined the differences in the physiological responses of different cowpea varieties to EBR and explored the physiological regulation function of EBR on cowpea plants under salt stress. Our findings will provide theoretical support for the use of EBR to relieve cowpea salt stress damage.

## 2 Materials and methods

### 2.1 Experiment materials

The 53 varieties of cowpea were used as the experiment materials (Table 1), all of which were provided by Hubei Province Engineering Research Center for Legume Plants.

**Table 1:** Fifty-three cowpea varieties

Variety number	Variety name	Geographical origin	Variety characteristics	Variety number	Variety name	Geographical origin	Variety characteristics
1	Qingtiao	Liaoning	Ramble, mid-ripe	28	Chaonenglinghang	Jiangxi	Ramble, precocity
2	Baiziqingtiao	Liaoling	Ramble, precocity	29	Sanjiang 100	Guangdong	Ramble, precocity
3	Chunqiuhong	Hubei	Ramble, precocity	30	Sanmei 8	Guangdong	Ramble, precocity
4	Yinyan	Hubei	Ramble, precocity	31	ID-0613	Hubei	Ramble, mid-ripe
5	Jiang Da Zi Jiang 1	Hubei	Ramble, precocity	32	WJ-60	Hubei	Ramble, mid-ripe
6	Bailongzaoshuai	Jiangxi	Ramble, precocity	33	Baidoujiao	Guangdong	Ramble, mid-ripe
7	Didou	America	Dwarf, late-maturing	34	Hongdoujiao	Guangdong	Ramble, mid-ripe
8	Ejiangdou 7	Hubei	Dwarf, late-maturing	35	Huadoujiao	Guangdong	Ramble, precocity
9	Liucui	Hubei	Ramble, mid-ripe	36	Ziqiujiang 6	Jiangxi	Ramble, mid-ripe
10	Huaguliang	Sichuan	Dwarf, mid-ripe	37	JD-0835	Hubei	Ramble, precocity
11	Xinwujia	Hubei	Ramble, late-maturing	38	Yuanzhongwujiadu	America	Dwarf, late-maturing
12	Baijinchanglong	Jiangxi	Ramble, precocity	39	Baipiduanjiang	Fujian	Ramble, late-maturing
13	Wufeng1	Hubei	Ramble, precocity	40	Didoujiao 0213	Ningxia	Ramble, precocity
14	Yibashuz	Hubei	Ramble, late-maturing	41	Hongshanyugu 2-13	Hubei	Ramble, late-maturing
15	Baiyulong	Shandong	Ramble, precocity	42	Wujiachunqiuhong	Jiangxi	Dwarf, mid-ripe
16	Sanchibaiyu	Shandong	Ramble, late-maturing	43	Cqingjiachang	Hubei	Ramble, mid-ripe
17	Yuanzhongguanjun	Shandong	Ramble, mid-ripe	44	A287-1	Hubei	Ramble, precocity
18	Yinjiangwang	Shandong	Ramble, precocity	45	A298 hong	Guangdong	Ramble, precocity
19	Huanglei	Liaoling	Ramble, mid-ripe	46	A298 hei	Guangdong	Ramble, precocity
20	901	Liaoling	Ramble, late-maturing	47	Zhanyanbaidoujiao	Fujian	Ramble, mid-ripe
21	Tiande 1	Sichuan	Ramble, late-maturing	48	Baipi	Fujian	Ramble, mid-ripe
22	902	Liaoling	Ramble, late-maturing	49	Huarong	Hubei	Ramble, mid-ripe
23	Yellow of qingtiao	Liaoling	Ramble, late-maturing	50	Huangjia	Hubei	Ramble, late-maturing
24	668	Liaoling	Ramble, mid-ripe	51	Lvguan	Jiangsu	Ramble, precocity
25	Chenbao 4	Hubei	Ramble, precocity	52	Yidianhong	Hong Kong	Ramble, precocity
26	Yipinduxiu	Jiangxi	Ramble, precocity	53	Changde cowpea	Hunan	Ramble, late-maturing
27	Zhecui 4	Zhejiang	Ramble, late-maturing				

### 2.2 Experiment design

The experiment was carried out in the plant incubator of Hubei Province Bean (Vegetable) Plant Engineering Technology Research Center from March to June 2022. Cowpea seeds were soaked at 45°C for 30 min, sown in plug trays with two cowpea seeds sown in each hole, and cultured at 28°C without light throughout the day. The culture conditions are shown in Table 2. After germination, when the seedlings grew to the stage of three leaves and one core, healthy seedlings with consistent growth status were selected and transplanted into hydroponic tanks. The hydroponic tank was 9 cm in diameter and10 cm in height. Three seedlings were cultured in each tank, and the culture medium was 1/2 Hoagland nutrient solution. After continuous culture for 7 days, EBR (0.1 μmol L^−1^, purchased from Sigma Company) was sprayed onto the seedlings once in the morning and once in the evening for 2 days. Immediately after EBR spraying, salt stress treatment was carried out. There were four treatments for each variety including CK treatment, 150 mmol L^−1^ NaCl treatment, 0.1 μmol L^−1^ EBR treatment, and 0.1 μmol L^−1^ EBR + 150 mmol L^−1^ NaCl treatment (Table 3).

**Table 2:** Incubator setting conditions

Time (h)	1	2	3	4	5	6
Temperature (℃)	16	16	16	16	17	18
Light intensity (lux)	0	0	0	0	0	3,000
Time (h)	7	8	9	10	11	12
Temperature (℃)	19	20	21	22	23	24
Light intensity (lux)	3,000	3,000	3,000	3,000	6,000	6,000
Time (h)	13	14	15	16	17	18
Temperature (℃)	25	24	23	22	21	20
Light intensity (lux)	6,000	6,000	3,000	3,000	3,000	3,000
Time (h)	19	20	21	22	23	24
Temperature (℃)	19	18	17	16	16	16
Light intensity (lux)	3,000	0	0	0	0	0

**Table 3:** Treatment

Number	Treatment
C	CK
N	150 mmol L^−1^ NaCl
E	0.1 μmol L^−1^ EBR
EN	0.1 μmol L^−1^ EBR + 150 mmol L^−1^ NaCl

### 2.3 Determination method

#### 2.3.1 Determination of photosynthetic parameters

Photosynthetic parameters were determined at 9:00–12:00 3 days after the NaCl stress. The light intensity was set at 1,200 μmol m^−2^ s^−1^, the CO_2_ volume fraction was set as 0.04%, and the temperature was set at 25°C. We measured the Pn (CO_2_ μmol m^−2^ s^−1^), Tr (H_2_O mmol m^−2^ s^−1^), Gs (H_2_O mol m^−2^ s^−1^), Ci (CO_2_ μmol mol^−1^), and other parameters of the functional leaves of each cowpea variety with LI-6400 portable plant photosynthesis instrument [9]. The measures were repeated three times, and the average value of three replicates was calculated.

#### 2.3.2 Fluorescence parameter measurement

Fluorescence parameter was measured at 9:00–12:00 3 days after the stress, the multi-channel continuous monitoring fluorescence instrument Monitoring-PAM (WALZ, Germany) was used to measure the chlorophyll fluorescence parameters excited by the laser after 15 min of dark adaptation. Y(II), qP, NPQ, and maximum photochemical quantum yield Fv/Fm were measured with three replicates, and the average was calculated [10].

#### 2.3.3 Measurement of spectral parameters

Spectral parameters were measured at 9:00–12:00 3 days after the stress, accompanied by the measurement of fluorescence parameters. The spectral reflectance of leaves of different varieties was measured using a plant leaf spectrometer (CI-710, United States). During the measurement, the leaves were placed flat, and the direction of the measured leaves was consistent. Three leaves were measured each time, and the average value was taken as the measured value of the reflectance spectrum parameters of the leaf. CRI1, ARI1, PRI, and PSRI were measured with three replicates, and the average value of three replicates was calculated [11].

#### 2.3.4 Determination of botanical traits

Botanical traits were determined 3 days after the stress, accompanied by the determination of photosynthetic parameters. The length and width of the leaves were measured with three replicates, and the average value was taken.

#### 2.3.5 Determination of photosynthetic pigments

The photosynthetic pigments of leaves were determined 3 days after the stress according to the method reported by Li et al. [12] with three replicates, and the average value was taken.

#### 2.3.6 Determination of photosynthetic enzyme

The photosynthetic enzyme was measured 3 days after the stress. Rubisco in leaves was measured according to the instruction in plant ribulose 1,5-biphosphate carboxylase kit (Nanjing Jiancheng) with three replicates. The average value of three replicates was taken.

### 2.4 Data processing

IBM SPSS Statistics 20 software was used to calculate the mean value and standard deviation and performed the principal component analysis (PCA) and the cluster analysis. The coefficient of variation (CV) was calculated by Excel 2016 software, and the correlation analysis was conducted with the Origin 2022 software. Hierarchical clustering method was adopted to classify cowpeas and the Euclidean distance was calculated.

## 3 Results

### 3.1 Performance and difference of exogenous EBR on photosynthetic traits of 53 cowpea varieties under NaCl stress

Table 4 shows that in CK, in 53 cowpea varieties, the average Pn was CO_2_ μmol m^−2^ s^−1^, the average of Gs was 0.469 H_2_O mol m^−2^ s^−1^, the average of Ci was 324.000 CO_2_ μmol mol^−1^, the average of Tr was 3.234 H_2_O mmol m^−2^ s^−1^, the average of Rubisco was 24.108 mg/g, the average of Chla was 0.558 mg/g, the average of Chlb was 0.199 mg/g, the average of Car was 0.113 mg/g, the average of total chlorophyll (Chl(a + b)) was 0.757 mg/g, the average leaf length was 7.509 cm, the average leaf width was 4.758 cm, the Y(II) was 0.623, the average of qP was 0.896, the average of NPQ was 0.044, the average of Fv/Fm was 0.703, the average ARI1 was 0.015, the average of CRI1 was 0.035, the average of PRI was 0.023, and the average of PSRI was 0.027. The CV of 19 photosynthetic traits of different varieties of cowpea was relatively large, ranging from 7.235 to 76.483%. Among them, the CV of NPQ was the largest, which was 76.483%, and that of qP was the smallest, which was 7.235%. The three CVs were ≤10%, and they were CV of Ci, qP, and Fv/Fm. Six CVs were between 10 and 20%, and they were CV of Pn, leaf length, leaf width, Y(II), ARI1, and CRI1. Twelve CVs were ＞20%, namely, CV of Gs, Tr, Rubisco, Chla, Chlb, Car, Chl(a + b), NPQ, PRI, and PSRI.

**Table 4:** Performance and difference of photosynthetic characters of cowpea varieties under CK

Properties	Mean value	Standard deviation	Maximum value	Minimum value	CV (%)
Pn (CO_2_ μmol m^−2^ s^−1^)	11.260	2.022	15.854	6.027	17.958
Gs (H_2_O mol m^−2^ s^−1^)	0.469	0.259	1.138	0.084	55.166
Ci (CO_2_ μmol mol^−1^)	324.000	24.920	361.118	259.374	7.691
Tr (H_2_O mmol m^−2^ s^−1^)	3.234	0.889	4.634	1.030	27.487
Rubisco (mg/g)	24.108	7.728	46.340	11.354	32.056
Chla (mg/g)	0.558	0.113	0.751	0.302	20.246
Chlb (mg/g)	0.199	0.042	0.281	0.109	20.945
Car (mg/g)	0.113	0.025	0.163	0.055	22.297
Chl(a + b) (mg/g)	0.757	0.153	1.031	0.411	20.272
Leaf length (cm)	7.509	0.752	10.100	5.867	10.018
Leaf width (cm)	4.758	0.559	6.367	3.733	11.739
Y(II)	0.623	0.072	0.745	0.421	11.587
qP	0.896	0.065	0.975	0.737	7.235
NPQ	0.044	0.034	0.182	0.009	76.483
Fv/Fm	0.703	0.061	0.819	0.506	8.647
ARI1	0.015	0.002	0.019	0.012	10.251
CRI1	0.035	0.006	0.046	0.018	16.918
PRI	0.023	0.008	0.046	0.004	36.383
PSRI	0.027	0.015	0.089	0.011	53.381

As shown in Table 5, under NaCl stress, in 53 cowpea varieties, the average Pn was 0.801 CO_2_ μmol m^−2^ s^−1^, the average Gs was 0.016 H_2_O mol m^−2^ s^−1^, the average Ci was 458.226 CO_2_ μmol mol^−1^, the average Tr was 0.203 H_2_O mmol m^−2^ s^−1^, the average Rubisco was 18.715 mg/g, the average Chla was 0.459 mg/g, the average Chlb was 0.168 mg/g, the average Car was 0.105 mg/g, the average Chl(a + b) was 0.627 mg/g, the average leaf length was 7.011 cm, the average leaf width was 4.558 cm, the average Y(II) was 0.690, the average qP was 0.918, the average NPQ was 0.043, the average Fv/Fm was 0.760, the average ARI1 was 0.015, the average CRI1 was 0.033, the average PRI was 0.029, and the average PSRI was 0.031. The CV of the 19 photosynthetic traits of different varieties of cowpea was relatively large, ranging from 5.503 to 86.645%. Pn exhibited the largest CV (86.645%), while qP displayed the smallest CV (5.503%). Three CVs were ≤10%, and they were CV of qP, Fv/Fm, and Y(II). Four CVs were between 10 and 20%, and they were CV of leaf length, leaf width, ARI1, and CRI1. Twelve CVs were >20%, namely, CV of Pn, Gs, Ci, Tr, Rubisco, Chla, Chlb, Car, Chl(a + b), NPQ, PRI, and PSRI.

**Table 5:** Performance and difference of photosynthetic characters of cowpea varieties under NaCl stress

Properties	Mean value	Standard deviation	Maximum value	Minimum value	CV (%)
Pn (CO_2_ μmol m^−2^ s^−1^)	0.801	0.694	3.419	0.024	86.645
Gs (H_2_O mol m^−2^ s^−1^)	0.016	0.013	0.065	0.000	77.915
Ci (CO_2_ μmol mol^−1^)	458.226	180.937	953.432	263.141	39.486
Tr (H_2_O mmol m^−2^ s^−1^)	0.203	0.149	0.769	0.005	73.511
Rubisco (mg/g)	18.715	7.410	40.972	6.007	39.593
Chla (mg/g)	0.459	0.117	1.023	0.299	25.488
Chlb (mg/g)	0.168	0.044	0.390	0.108	26.103
Car (mg/g)	0.105	0.022	0.206	0.064	20.814
Chl(a + b) (mg/g)	0.627	0.160	1.413	0.407	25.525
Leaf length (cm)	7.011	0.851	8.833	5.233	12.132
Leaf width (cm)	4.558	0.547	6.367	3.400	11.992
Y(II)	0.690	0.047	0.782	0.520	6.755
qP	0.918	0.051	0.983	0.749	5.503
NPQ	0.043	0.032	0.188	0.014	74.637
Fv/Fm	0.760	0.053	0.936	0.609	7.001
ARI1	0.015	0.002	0.019	0.012	10.357
CRI1	0.033	0.005	0.043	0.018	16.140
PRI	0.029	0.008	0.048	0.013	29.043
PSRI	0.031	0.014	0.076	0.005	45.553

As shown in Table 6, under EBR treatment, in 53 cowpea varieties, the average Pn was 10.909 μmol m^−2^ s^−1^, the average Gs was 0.453 H_2_O mol m^−2^ s^−1^, the average Ci was 321.971 CO_2_ μmol mol^−1^, the average Tr was 3.105 H_2_O mmol m^−2^ s^−1^, the average Rubisco was 29.137 mg/g, the average Chla was 0.577 mg/g, the average Chlb was 0.208 mg/g, the average Car was 0.114 mg/g, the average Chl(a + b) was 0.785 mg/g, the average leaf length was 7.289 cm, the average leaf width was 4.733 cm, the average Y(II) was 0.623, the average qP was 0.899, the average NPQ was 0.050, the average Fv/Fm was 0.704, the average ARI1 was 0.015, the average CRI1 was 0.034, the average PRI was 0.025, and the average PSRI was 0.027. The CVs of the 19 photosynthetic traits of different varieties of cowpea were relatively large, ranging from 5.805 to 103.782%. Among them, the CV of NPQ was the highest (103.782%), while the CV of qP was the smallest (5.805%). Four CVs were ≤10%, and they were CV of Ci, qP, Y(II), and Fv/Fm. Six CVs were between 10 and 20%, and they were CV of Pn, Chla, leaf length, leaf width, ARI1, and CRI1. Nine CVs were >20%, namely, CV of Gs, Tr, Rubisco, Chlb, Car, Chl(a + b), NPQ, PRI, and PSRI.

**Table 6:** Performance and difference of photosynthetic characters of cowpea varieties under EBR treatment

Properties	Mean value	Standard deviation	Maximum value	Minimum value	CV (%)
Pn (CO_2_ μmol m^−2^ s^−1^)	10.909	1.439	14.352	7.797	13.193
Gs (H_2_O mol m^−2^ s^−1^)	0.453	0.262	1.097	0.074	57.679
Ci (CO_2_ μmol mol^−1^)	321.971	28.595	361.450	249.686	8.881
Tr (H_2_O mmol m^−2^ s^−1^)	3.105	0.909	4.670	0.767	29.272
Rubisco (mg/g)	29.137	8.663	55.212	14.105	29.733
Chla (mg/g)	0.577	0.115	0.786	0.343	19.995
Chlb (mg/g)	0.208	0.048	0.338	0.126	23.337
Car (mg/g)	0.114	0.024	0.164	0.065	21.478
Chl(a + b) (mg/g)	0.785	0.162	1.089	0.468	20.665
Leaf length (cm)	7.289	0.735	8.900	5.400	10.077
Leaf width (cm)	4.733	0.528	6.200	3.800	11.159
Y(II)	0.623	0.044	0.692	0.511	7.113
qP	0.899	0.052	0.981	0.772	5.805
NPQ	0.050	0.052	0.361	0.014	103.782
Fv/Fm	0.704	0.053	0.795	0.543	7.468
ARI1	0.015	0.002	0.019	0.012	11.296
CRI1	0.034	0.006	0.049	0.017	16.138
PRI	0.025	0.010	0.060	0.006	39.963
PSRI	0.027	0.015	0.092	0.011	57.264

It can be seen from Table 7, under EBR treatment and NaCl stress, in 53 cowpea varieties that the average Pn was 0.870 CO_2_ μmol m^−2^ s^−1^, the average Gs was 0.015 H_2_O mol m^−2^ s^−1^, the average Ci was 435.247 CO_2_ μmol mol^−1^, the average Tr was 0.186 H_2_O mmol m^−2^ s^−1^, the average Rubisco was 21.785 mg/g, the average Chla was 0.431 mg/g, the average Chlb was 0.159 mg/g, the average Car was 0.099 mg/g, the average Chl(a + b) was 0.590 mg/g, the average leaf length was 7.194 cm, the average leaf width was 4.587 cm, the average Y(II) was 0.696, the average qP was 0.922, the average NPQ was 0.035, the average Fv/Fm was 0.761, the average ARI1 was 0.015, the average CRI1 was 0.034, the average PRI was 0.029, and the average PSRI was 0.025. The CVs of the 19 photosynthetic traits of different varieties of cowpea were relatively large, ranging from 5.745 to 71.130%. Among them, the CV of NPQ was the largest, which was 71.130%, while the CV of qP was the smallest, which was 5.745%. Three CVs were ≤10%, and they were CV of Y(II), qP, and Fv/Fm. Five CVs were between 10 and 20%, which were CV of Car, leaf length, leaf width, ARI1, and CRI1. Eleven CVs were >20%, namely, CV of Pn, Ci, Gs, Tr, Rubisco, Chla, Chlb, Chl(a + b), NPQ, PRI, and PSRI.

**Table 7:** Performance and difference of photosynthetic characters of cowpea varieties under EBR treatment and NaCl stress

Properties	Mean value	Standard deviation	Maximum value	Minimum value	CV (%)
Pn (CO_2_ μmol m^−2^ s^−1^)	0.870	0.600	3.096	0.015	69.021
Gs (H_2_O mol m^−2^ s^−1^)	0.015	0.009	0.035	0.001	58.429
Ci (CO_2_ μmol mol^−1^)	435.247	176.305	992.758	222.016	40.507
Tr (H_2_O mmol m^−2^ s^−1^)	0.186	0.098	0.416	0.017	52.684
Rubisco (mg/g)	21.785	7.585	40.250	8.941	34.817
Chla (mg/g)	0.431	0.089	0.723	0.272	20.703
Chlb (mg/g)	0.159	0.034	0.266	0.095	21.699
Car (mg/g)	0.099	0.018	0.136	0.067	18.552
Chl(a + b) (mg/g)	0.590	0.123	0.989	0.367	20.768
Leaf length (cm)	7.194	0.782	9.367	5.467	10.875
Leaf width (cm)	4.587	0.548	5.867	2.933	11.953
Y(II)	0.696	0.064	0.774	0.447	9.236
qP	0.922	0.053	0.978	0.766	5.745
NPQ	0.035	0.025	0.162	0.010	71.130
Fv/Fm	0.761	0.054	0.866	0.590	7.095
ARI1	0.015	0.002	0.019	0.011	12.444
CRI1	0.034	0.005	0.046	0.017	15.841
PRI	0.029	0.009	0.046	0.007	29.744
PSRI	0.025	0.014	0.093	0.004	57.535

### 3.2 Correlation analysis of exogenous EBR on photosynthetic traits of 53 cowpea varieties under NaCl stress

**Figure 1:**
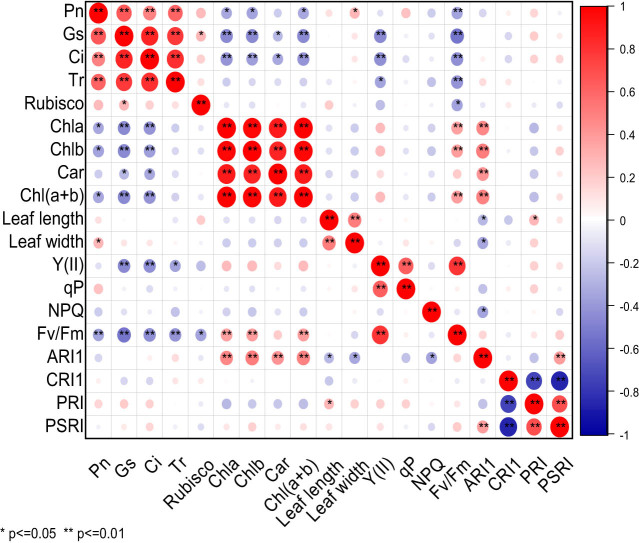
Correlation analysis of photosynthetic characters of cowpea varieties under CK. ** Indicates significant correlation at 0.01 level, while * indicates significant correlation at 0.05 level. The red balls in the graph represent a positive correlation between two indicators, while the blue balls represent a negative correlation. The deeper color or larger size of the balls represents a stronger correlation.

Figure 1 shows that in CK, significant differences in the correlation were observed among 19 photosynthetic traits of cowpea varieties. Pn was extremely significantly positively correlated with Gs, Ci, Tr, and significantly positively correlated with leaf width, but extremely significantly negatively correlated with Fv/Fm, and significantly negatively correlated with Chla, Chlb, and Chl(a + b). Gs was significantly positively correlated with Rubisco, but extremely significantly negatively correlated with Chla, Chlb, Chl(a + b), and significantly negatively correlated with Car. Ci was extremely significantly negatively correlated with Chla, Chlb, Chl(a + b), Y(II), Fv/Fm, and significantly negatively correlated with Car. Tr exhibited an extremely significant negative correlation with Fv/Fm, and significant negative correlation with Y(II). Rubisco displayed a significant negative correlation with Fv/Fm. Chla showed an extremely significant positive correlation with Chlb, Car, Chl(a + b), Fv/Fm, and ARI1. Chlb was extremely significantly positively correlated with Car, Chl(a + b), Fv/Fm, and ARI1. Car exhibited an extremely significant positive correlation with Chl(a + b) and ARI1. Chl(a + b) showed an extremely significant positive correlation with Fv/Fm and ARI1. Leaf length was extremely significantly positively correlated with leaf width, and significantly positively correlated with PRI, but significantly negatively correlated with ARI1. Leaf width was significantly negatively correlated with ARI1. Y(II) was extremely significantly positively correlated with qP and Fv/Fm. NPQ exhibited a significant negative correlation with ARI1. ARI1 displayed a highly significant positive correlation with PSRI. CRI1 presented a significant negative correlation with PRI and PSRI. There was a very significant positive correlation between PRI and PSRI. Under CK treatment, Pn displayed an extremely significant positive correlation with Gs, Ci, Tr, and significant positive correlation with leaf width, but an extremely significant negative correlation with Fv/Fm, and significant negative correlation with Chla, Chlb, and Chl(a + b).

**Figure 2:**
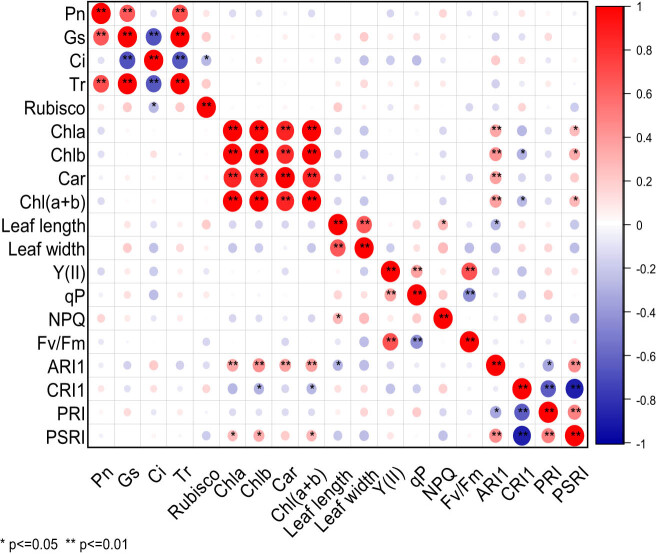
Correlation analysis of photosynthetic characters of cowpea varieties under NaCl stress. ** Indicates significant correlation at 0.01 level, while * indicates significant correlation at 0.05 level. The red balls in the graph represent a positive correlation between two indicators, while the blue balls represent a negative correlation. The deeper color or larger size of the balls represents a stronger correlation.

It can be seen from Figure 2 that under NaCl stress, there were significant differences in the correlation of 19 photosynthetic traits of cowpea varieties. Pn exhibited highly significant positive correlation with Gs and Tr. Gs was extremely significantly positively correlated with Tr, but extremely significantly negatively correlated with Ci. Ci was extremely significantly negatively correlated with Tr, and significantly negatively correlated with rubisco. Chla was highly significantly positively correlated with Chlb, Car, Chl(a + b), and ARI1, and it was significantly positively correlated with PSRI. Chlb exhibited an extremely significant positive correlation with Car, Chl(a + b), and ARI1, a significant positive correlation with PSRI, but a significant negative correlation with CRI1. Car displayed a significant positive correlation with Chl(a + b) and ARI1. Chl(a + b) presented a highly significant positive correlation with ARI1, and a significant positive correlation with PSRI, but a significant negative correlation with CRI1. Leaf length was extremely significantly positively correlated with leaf width, significantly positively correlated with NPQ, and significantly negatively correlated with ARI1. Y(II) was extremely significantly positively correlated with qP and Fv/Fm. qP was extremely significantly negatively correlated with Fv/Fm. ARI1 was highly significantly positively correlated with PSRI, but significantly negatively correlated with PRI. CRI1 was significantly negatively correlated with PRI and PSRI. There was a very significant positive correlation between PRI and PSRI. Under NaCl stress, Pn was highly significantly positively correlated with Gs and Tr, but extremely significantly negatively correlated with Ci.

**Figure 3:**
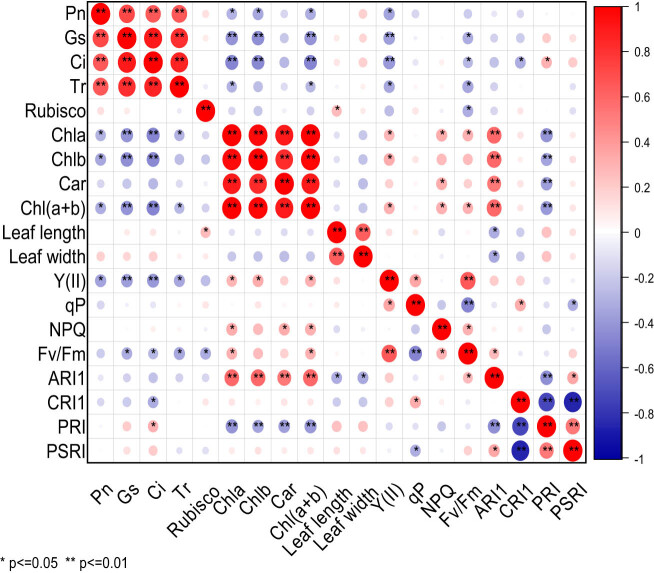
Correlation analysis of photosynthetic characters of cowpea varieties under EBR treatment. ** Indicates significant correlation at 0.01 level, while * indicates significant correlation at 0.05 level. The red balls in the graph represent a positive correlation between two indicators, while the blue balls represent a negative correlation. The deeper color or larger size of the balls represents a stronger correlation.

As shown in Figure 3, under the EBR treatment, there were significant differences in the correlation of 19 photosynthetic traits of cowpea varieties. Pn was highly significantly positively correlated with Gs, Ci, and Tr, but significantly negatively correlated with Chla, Chlb, Chl(a + b), and Y(II). Gs was extremely significantly positively correlated with Ci and Tr, but extremely significantly negatively correlated with Chla, Chlb, Chl(a + b), and significantly negatively correlated with Fv/Fm. There was a highly significant positive correlation between Ci and Tr, and a significant positive correlation between Ci and PRI, but a highly significant negative correlation between Ci and Chla, Chlb, Chl(a + b), or Y(II), and a significant negative correlation between Ci and Fv/Fm or CRI1. Tr was significantly negatively correlated with Chla, Chl(a + b), Fv/Fm, and Y(II). Rubisco was significantly positively correlated with leaf length, but significantly negatively correlated with Fv/Fm. A highly significant positive correlation was observed between Chla and Chlb, Car, Chl(a + b), or ARI1, and a significant positive correlation between Chla and Fv/Fm, Y(II), or NPQ, but an extremely significant negative correlation between Chla and PRI. Chlb was highly significantly positively correlated with Car, Chl(a + b), and ARI1, and significantly positively correlated with Y(II), but extremely significantly negatively correlated with PRI. Car displayed a highly significant positive correlation with Chl(a + b) and ARI1, a significant positive correlation with NPQ, and an extremely significant negative correlation with PRI. Chl(a + b) exhibited a highly significant positive correlation with ARI1, a significant positive correlation with Fv/Fm, Y(II), and NPQ, but a highly significant negative correlation with PRI. Leaf length was highly significantly positively correlated with leaf width, and significantly negatively correlated with ARI1. Leaf width was significantly negatively correlated with ARI1. Y(II) was highly significantly positively correlated with Fv/Fm and significantly positively correlated with qP. There was a significant positive correlation between qP and CRI1, and an extremely significant negative correlation with Fv/Fm, but a significant negative correlation with PSRI. A significant positive correlation was observed between NPQ and Fv/Fm, between Fv/Fm and ARI1, between ARI1 and PSRI, whereas an extremely significant negative correlation between ARI1and PRI. A significant negative correlation was found between CRI1 and PRI or PSRI. There was a very significant positive correlation between PRI and PSRI. Under EBR treatment, Pn was extremely significantly positively correlated with Gs, Ci, Tr, and significantly negatively correlated with Chla, Chlb, Chl(a + b), and Y(II).

**Figure 4:**
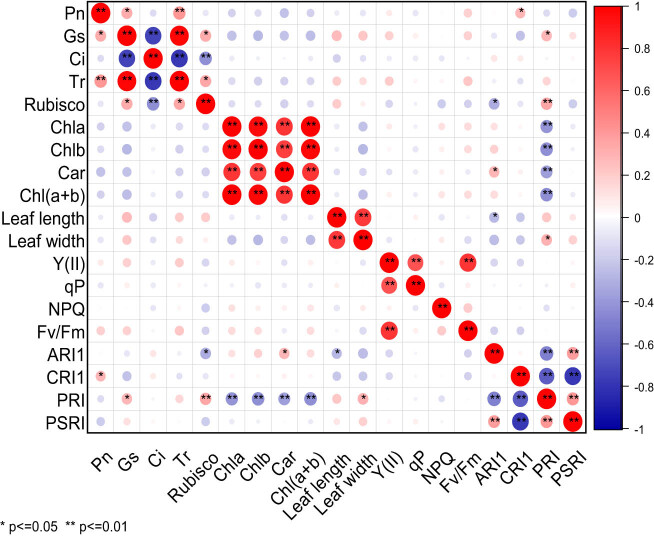
Correlation analysis of photosynthetic characters of cowpea varieties under EBR treatment and NaCl stress. ** Indicates significant correlation at 0.01 level, while * indicates significant correlation at 0.05 level. The red balls in the graph represent a positive correlation between two indicators, while the blue balls represent a negative correlation. The deeper color or larger size of the balls represents a stronger correlation.

It can be seen from Figure 4 that there were significant differences in the correlation among 19 photosynthetic traits of cowpea varieties under EBR treatment and NaCl stress. Pn was extremely significantly positively correlated with Tr, and significantly positively correlated with Gs and CRI1. Gs was extremely significantly positively correlated with Tr, significantly positively correlated with rubisco and PRI, and extremely significantly negatively correlated with Ci. There was an extremely significant negative correlation between Ci and Tr or rubisco, but a significant positive correlation between Tr and rubisco. A highly significant positive correlation was observed between rubisco and PRI, but a significant negative correlation between rubisco and ARI1. Chla was highly significantly positively correlated with Chlb, Car, Chl(a + b), and extremely significantly negatively correlated with PRI. Chlb was highly significantly positively correlated with Car and Chl(a + b), and extremely significantly negatively correlated with PRI. Car displayed an extremely significant positive correlation with Chl(a + b), and a significant positive correlation with ARI1, but an extremely significant negative correlation with PRI. Chl(a + b) exhibited an extremely significant negative correlation with PRI. Leaf length was highly significantly positively correlated with leaf width, and significantly negatively correlated with ARI1. Leaf width and PRI were significantly positively correlated. There was a highly significant positive correlation between Y(II) and qP or Fv/Fm, between ARI1and PSRI, but an extremely significant negative correlation between ARI1 and PRI. CRI1 displayed a significant negative correlation with PRI and PSRI. There was an extremely significant positive correlation between PRI and PSRI. Under EBR treatment and NaCl stress, Pn was extremely significantly positively correlated with Tr, but significantly positively correlated with Gs and CRI1.

The above correlation analysis results of the 19 photosynthetic traits of cowpea under four treatments revealed that Pn and Tr were extremely significantly positively correlated under all treatments, and Pn and Gs were significantly positively correlated. Pn and Ci were extremely significantly positively correlated under C and E treatments in the control group, whereas they were negatively correlated under N and EN treatments, but not significantly. Pn was significantly negatively correlated with Chla, Chlb, Chl(a + b) in the control group under C and E treatments, but they were negatively correlated under N and EN treatments without significance.

### 3.3 PCA of photosynthetic traits of 53 cowpea varieties after applying exogenous EBR under NaCl stress

**Figure 5:**
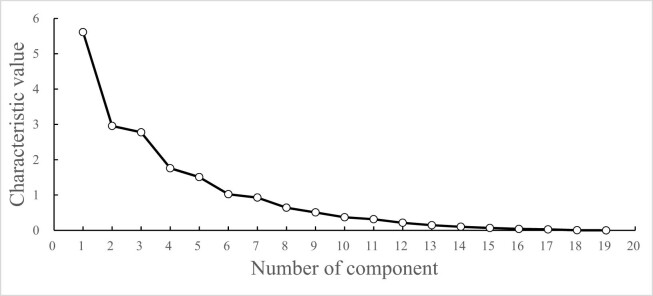
Scree plot of PCA of photosynthetic characteristics of cowpea varieties under CK.

As can be seen from Figure 5, with the increase in the number of principal components, the eigenvalues gradually decrease. The eigenvalues of the first six principal components are all greater than 1, and the connecting lines are steeper, indicating that the first six principal components contribute the most to explaining the variables. Therefore, the first six principal components are extracted.

In CK, based on 19 photosynthetic traits of 53 cowpea varieties, the eigenvectors and contribution rates of each principal component were calculated (Table 8). As for the first principal component (PC1), its eigenvalue was 5.613, and the contribution rate was 29.543%. With Pn, Gs, Ci, Chla, Chlb, Car, Chl(a + b), and Fv/Fm serving as the main indicators, the eigenvectors were −0.592, −0.742, −0.690, 0.881, 0.869, 0.749, 0.885, and 0.622, respectively. The PC1 mainly reflected photosynthetic factors. The eigenvalue of the second principal component (PC2) was 2.956, and its contribution rate was 15.558%. With Tr and ARI1 as the main indicators, the eigenvectors were 0.658 and 0.559, respectively, and the vectors were 0.658 and 0.559. The PC2 mainly reflected the transpiration factor. The eigenvalue of the third principal component (PC3) was 2.776, and the contribution rate was 14.611%. With PRI, PSRI, and CRI1 as the main indicators, the eigenvectors were 0.759, 0.922, and −0.855, and this principal component mainly reflected the photochemical reflection factor. The eigenvalue of the fourth principal component (PC4) was 1.759, and the contribution rate was 9.259%. With qP and Y(II) as the main indicators, the eigenvectors were 0.711 and 0.554, respectively, and the PC4 mainly reflected qP factor. The eigenvalue of the fifth principal component (PC5) was 1.508, and its contribution rate was 7.938%. With the leaf length and leaf width as the main indicators, the eigenvectors were 0.653 and 0.561, respectively. The PC5 mainly reflected the leaf length and width factor. The eigenvalue of the six principal components was 1.024, and its contribution rate was 5.390%. With NPQ and Rubisco as the main indicators, the eigenvectors were 0.419 and 0.507, respectively, this principal component mainly reflected the photosynthetic enzyme factor. In CK treatment, the photosynthetic traits of cowpea varieties were mainly assigned into six principal components with a contribution rate of 82.298%, and these six principle components could replace 19 photosynthetic traits to evaluate 53 cowpea varieties.

**Table 8:** PCA of photosynthetic characters of cowpea varieties under CK

Properties	PC1	PC2	PC3	PC4	PC5	PC6
Pn	−0.592	0.256	0.165	0.581	0.014	0.011
Gs	−0.742	0.470	0.291	0.121	−0.058	0.063
Ci	−0.690	0.471	0.323	0.014	−0.126	−0.043
Tr	−0.520	0.658	0.168	0.271	−0.161	−0.119
Rubisco	−0.259	0.269	−0.083	0.186	0.397	0.507
Chla	0.881	0.342	0.104	0.139	0.206	0.037
Chlb	0.869	0.337	0.184	0.100	0.167	−0.001
Car	0.749	0.407	0.113	0.164	0.259	0.212
Chl(a + b)	0.885	0.343	0.126	0.130	0.197	0.028
Leaf length	−0.179	−0.337	0.234	0.205	0.653	−0.249
Leaf width	−0.304	−0.259	0.116	0.284	0.561	−0.438
Y(II)	0.483	−0.516	0.167	0.554	−0.359	0.013
qP	−0.024	−0.389	0.018	0.711	−0.213	0.372
NPQ	−0.118	−0.333	−0.266	−0.321	0.194	0.419
Fv/Fm	0.622	−0.409	0.184	0.134	−0.296	−0.242
ARI1	0.434	0.559	0.304	−0.124	−0.277	−0.128
CRI1	0.037	0.208	−0.855	0.318	−0.097	−0.147
PRI	−0.253	−0.435	0.759	−0.009	0.016	0.176
PSRI	0.106	−0.086	0.922	−0.232	−0.054	0.066
Characteristic value	5.613	2.956	2.776	1.759	1.508	1.024
Percentage	29.543	15.558	14.611	9.259	7.938	5.390
Cumulative contribution rate (%)	29.543	45.101	59.711	68.970	76.908	82.298

**Figure 6:**
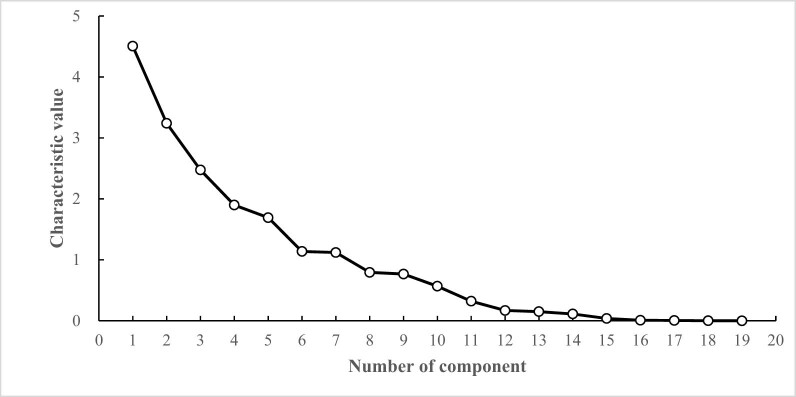
Scree plot of PCA of photosynthetic characteristics of cowpea varieties under NaCl stress.

As can be seen from Figure 6, with the increase in the number of principal components, the eigenvalues gradually decrease. The eigenvalues of the first seven principal components are all greater than 1, and the connecting lines are steeper, indicating that the first seven principal components contribute the most to explaining the variables. Therefore, the first seven principal components are extracted.

Under NaCl stress, based on 19 photosynthetic traits of 53 cowpea varieties, the eigenvectors and contribution rates of each principal component were calculated (Table 9). The eigenvalue of the PC1 was 4.505, and the contribution rate was 23.713%. With Chla, Chlb, Car, Chl(a + b), and ARI1 as the main indicators, the eigenvectors were 0.904, 0.919, 0.796, 0.913, and 0.575, respectively. The PC1 mainly reflected the photosynthetic pigment factor. The eigenvalue of the PC2 was 3.240, and its contribution rate was 17.051%. With Pn, Gs, Ci, and Tr as the main indicators, the eigenvectors were 0.543, 0.922, −0.707, and 0.893, respectively. PC2 mainly reflected the photosynthesis factor. The eigenvalue of the PC3 was 2.474, and its contribution rate was 13.02%. Taking PRI, PSRI, and CRI1 as the main indicators, the eigenvectors were −0.704, −0.674, and 0.759, respectively, and PC3 mainly reflected the photochemical reflection factor. The eigenvalue of the PC4 was 1.899, and its contribution rate was 9.994%. With the leaf length, leaf width, and Fv/Fm as the main indicators, the eigenvectors were 0.521, 0.577, −0.628, and PC4 mainly reflected the leaf length and width factor. The eigenvalue of the PC5 was 1.692, and the contribution rate was 8.907%. Taking Y(II) as the main index, the eigenvector was 0.762, PC5 mainly represented the Y(II) factor. The eigenvalue of the sixth principal component (PC6) was 1.137, and its contribution rate was 5.984%. Taking qP as the main index, the eigenvector was −0.669, and PC6 mainly indicated the qP factor. The eigenvalue of the seventh principal component (PC7) was 1.12, and its contribution rate was 5.894%, taking NPQ and Rubisco as the main indicators, and the eigenvectors were 0.555 and −0.577, respectively, and PC7 mainly represented the photosynthetic enzyme factor. Under NaCl stress, the photosynthetic traits of cowpea varieties were mainly assigned into seven principal components with a cumulative contribution rate of 84.564%.

**Table 9:** PCA of photosynthetic characters of cowpea varieties under NaCl stress

Properties	PC1	PC2	PC3	PC4	PC5	PC6	PC7
Pn	−0.242	0.543	0.156	−0.375	−0.394	−0.079	0.298
Gs	−0.180	0.922	0.049	−0.255	−0.108	0.007	0.058
Ci	0.233	−0.707	0.035	0.061	−0.166	0.063	0.270
Tr	−0.205	0.893	0.072	−0.326	−0.117	0.007	0.044
Rubisco	−0.114	0.310	0.319	0.093	0.016	0.061	−0.577
Chla	0.904	0.246	0.223	0.046	0.185	0.087	−0.043
Chlb	0.919	0.198	0.213	0.053	0.131	0.108	0.042
Car	0.796	0.259	0.344	0.130	0.053	0.053	0.039
Chl(a + b)	0.913	0.234	0.221	0.048	0.171	0.093	−0.020
Leaf length	−0.381	0.205	0.195	0.521	0.325	0.311	0.220
Leaf width	−0.402	0.256	0.245	0.577	0.088	0.344	0.286
Y(II)	−0.008	0.152	−0.506	−0.209	0.762	−0.176	−0.043
qP	−0.058	0.316	−0.147	0.526	0.248	−0.669	−0.162
NPQ	−0.286	0.128	0.236	0.115	0.368	−0.323	0.555
Fv/Fm	0.017	−0.109	−0.327	−0.628	0.574	0.323	0.144
ARI1	0.575	−0.158	0.117	−0.099	−0.223	−0.362	0.290
CRI1	−0.416	−0.304	0.759	−0.213	0.116	−0.062	−0.053
PRI	−0.074	0.282	−0.704	0.388	−0.195	0.236	−0.019
PSRI	0.524	0.153	−0.674	0.107	−0.280	−0.019	0.201
Characteristic value	4.505	3.240	2.474	1.899	1.692	1.137	1.120
Percentage	23.713	17.051	13.02	9.994	8.907	5.984	5.894
Cumulative contribution rate (%)	23.713	40.764	53.784	63.778	72.685	78.669	84.564

**Figure 7:**
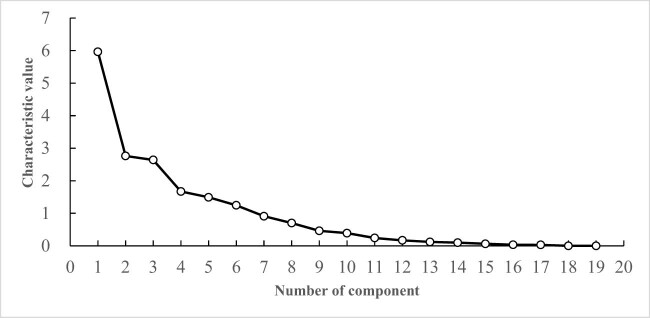
Scree plot of PCA of photosynthetic characteristics of cowpea varieties under EBR treatment.

As shown in Figure 7, with the increase in the number of principal components, the eigenvalues gradually decrease. The eigenvalues of the first six principal components are all greater than 1, and the connecting line is relatively steep, indicating that the first six principal components contribute the most to explaining the variables. Therefore, the first six principal components are extracted.

Under the EBR treatment, based on 19 photosynthetic traits of 53 cowpea varieties, the eigenvectors and contribution rates of each principal component were calculated (Table 10). The eigenvalue of the PC1 was 5.964, and its contribution rate was 31.391%. With Pn, Gs, Ci, Tr, Chla, Chlb, Car, Chl(a + b), and ARI1 as the main indicators, the eigenvectors were −0.545, −0.694, −0.732, −0.557, 0.889, 0.875, 0.752, 0.894, and 0.612, and PC1 mainly reflected the photosynthesis factor. The eigenvalue of the PC2 was 2.763, and its contribution rate was 14.544%. With PSRI and CRI1 as the main indicators, the eigenvectors were 0.812 and −0.744, PC2 mainly reflected the photochemical reflection factor. The eigenvalue of the PC3 was 2.641, and the contribution rate was 13.901%. With PRI and Tr as the main indicators, the eigenvectors were −0.587 and 0.666, and PC3 mainly represented the transpiration factor. The eigenvalue of the PC4 was 1.671, and its contribution rate was 8.796%. With the leaf length and leaf width as the main indicators, the eigenvectors were 0.761 and 0.632, respectively, and PC4 mainly represented the leaf length and width factor. The eigenvalue of the PC5 was 1.493, and its contribution rate was 7.856%. With Fv/Fm as the main index, the eigenvector was 0.714, and PC5 mainly reflected the maximum photochemical yield factor. The eigenvalue of the PC6 was 1.246, and its contribution rate was 6.558%. With NPQ, qP, Y(II), and Rubisco as the main indicators, the eigenvectors were −0.415, 0.526, 0.525, and −0.576, respectively, and PC6 mainly represented the photosynthetic enzyme factor. Under EBR treatment, the photosynthetic traits of cowpea varieties were summarized into six principal components, with a cumulative contribution rate of 83.046%.

**Table 10:** PCA of photosynthetic characters of cowpea varieties under EBR treatment

Properties	PC1	PC2	PC3	PC4	PC5	PC6
Pn	−0.545	0.318	0.491	0.112	0.162	−0.049
Gs	−0.694	0.377	0.47	0.143	0.069	0.137
Ci	−0.732	0.443	0.379	0.109	0.096	0.147
Tr	−0.557	0.242	0.666	0.134	0.129	0.243
Rubisco	−0.206	−0.243	0.046	0.336	−0.332	−0.576
Chla	0.889	0.28	0.184	0.223	−0.085	−0.063
Chlb	0.875	0.271	0.157	0.216	−0.14	0.063
Car	0.752	0.321	0.334	0.235	−0.146	−0.076
Chl(a + b)	0.894	0.28	0.178	0.223	−0.102	−0.026
Leaf length	−0.222	0.037	−0.41	0.761	0.104	−0.135
Leaf width	−0.343	0.095	−0.337	0.632	0.274	−0.098
Y(II)	0.492	−0.16	−0.308	0.233	0.421	0.525
qP	0.067	−0.479	0.154	0.458	−0.35	0.526
NPQ	0.26	0.263	0.285	0.026	0.413	−0.415
Fv/Fm	0.449	0.269	−0.354	−0.124	0.714	0.004
ARI1	0.612	0.369	0.209	−0.172	−0.121	0.149
CRI1	0.273	−0.744	0.476	0.057	0.253	−0.025
PRI	−0.492	0.341	−0.587	0.003	−0.23	0.116
PSRI	−0.03	0.812	−0.41	−0.06	−0.264	0.095
Characteristic value	5.964	2.763	2.641	1.671	1.493	1.246
Percentage	31.391	14.544	13.901	8.796	7.856	6.558
Cumulative contribution rate (%)	31.391	45.936	59.837	68.632	76.488	83.046

**Figure 8:**
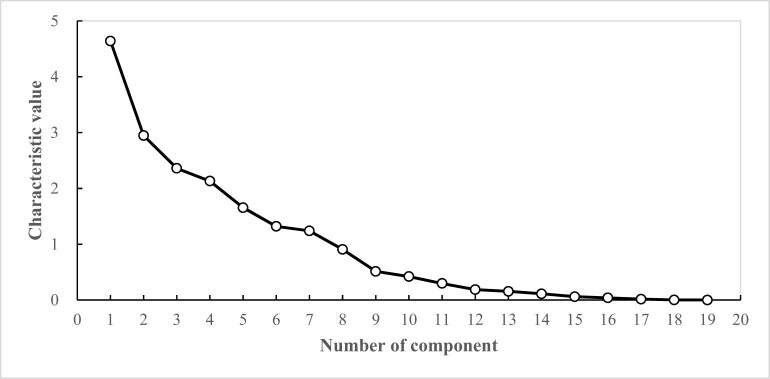
Scree plot of PCA of photosynthetic characteristics of cowpea varieties under EBR treatment and NaCl stress.

As shown in Figure 8, with the increase in the number of principal components, the eigenvalues gradually decrease. The eigenvalues of the first seven principal components are all greater than 1, and the connecting line is relatively steep, indicating that the first seven principal components contribute the most to explaining the variables. Therefore, the first seven principal components are extracted.

Under EBR treatment and NaCl stress, the eigenvectors and contribution rates of each principal component were calculated, based on 19 photosynthetic traits of 53 cowpea varieties (Table 11). The eigenvalue of PC1 was 4.639, and its contribution rate was 24.418%. With Chla, Chlb, Car, Chl(a + b), and PRI as the main indicators, the eigenvectors were 0.824, 0.828, 0.760, 0.833, −0.664, respectively, and PC1 mainly represented the photosynthetic pigment factor. The eigenvalue of PC2 was 2.948, and its contribution rate was 15.517%. With Gs, Ci, and Tr as the main indicators, the eigenvectors were 0.659, −0.719, 0.714, respectively, PC2 mainly reflected the transpiration factor. The eigenvalue of PC3 was 2.360, and its contribution rate was 12.419%. With Pn, PSRI, and CRI1 as the main indicators, the eigenvectors were −0.621, 0.705, and −0.760, respectively, PC3 mainly represented the photosynthetic rate factor. The eigenvalue of PC4 was 2.131, and its contribution rate was 11.215%. With Y(II) and Fv/Fm as the main indicators, the eigenvectors were 0.783 and 0.638, respectively, and PC4 mainly represented the Y(II) factor. The eigenvalue of PC5 was 1.655, and the contribution rate was 8.709%. With ARI1 as the main index, the eigenvectors were −0.767, and PC5 mainly reflected the anthocyanin reflection factor. The eigenvalue of PC6 was 1.320, and its contribution rate was 6.945%. With the leaf length, leaf width, and Rubisco as the main indicators, the eigenvectors were 0.471, 0.621, −0.511, respectively, and PC6 mainly represented the leaf length and width factor. The eigenvalue of the PC7 was 1.239, and the contribution rate was 6.519%. With NPQ and qP as the main indicators, the eigenvectors were −0.466 and 0.691, respectively, and PC7 mainly reflected the qP factor. Under EBR treatment and NaCl stress, the photosynthetic traits of cowpea varieties could be assigned into seven principal components, with a cumulative contribution rate of 85.742%.

**Table 11:** PCA of photosynthetic characters of cowpea varieties under EBR treatment and NaCl stress

Properties	PC1	PC2	PC3	PC4	PC5	PC6	PC7
Pn	−0.187	0.168	−0.621	0.034	−0.231	0.251	−0.160
Gs	−0.618	0.659	−0.154	−0.155	−0.265	0.013	0.039
Ci	0.294	−0.719	0.152	0.381	0.135	0.101	−0.106
Tr	−0.518	0.714	−0.278	−0.185	−0.267	0.027	0.016
Rubisco	−0.391	0.214	0.016	−0.504	0.220	−0.511	0.021
Chla	0.824	0.473	0.177	−0.121	0.117	−0.030	−0.047
Chlb	0.828	0.434	0.182	−0.123	0.081	−0.043	−0.054
Car	0.760	0.291	0.241	−0.219	−0.08	0.026	0.092
Chl(a + b)	0.833	0.466	0.180	−0.123	0.109	−0.034	−0.049
Leaf length	−0.374	0.235	0.395	−0.254	0.389	0.471	0.241
Leaf width	−0.453	0.055	0.426	−0.235	0.275	0.621	0.120
Y(II)	−0.065	0.501	−0.125	0.783	0.275	−0.039	0.162
qP	−0.104	0.205	−0.033	0.513	0.224	−0.106	0.691
NPQ	0.119	0.168	−0.076	0.115	−0.030	0.466	−0.466
Fv/Fm	0.006	0.502	−0.143	0.638	0.174	0.043	−0.385
ARI1	0.374	−0.083	−0.011	0.065	−0.767	0.161	0.326
CRI1	0.311	−0.234	−0.760	−0.250	0.236	0.183	0.179
PRI	−0.664	−0.008	0.49	0.051	0.090	−0.281	−0.319
PSRI	−0.168	0.08	0.705	0.306	−0.565	0.046	0.048
Characteristic value	4.639	2.948	2.360	2.131	1.655	1.320	1.239
Percentage	24.418	15.517	12.419	11.215	8.709	6.945	6.519
Cumulative contribution rate (%)	24.418	39.934	52.354	63.569	72.278	79.223	85.742

### 3.4 Cluster analysis of photosynthetic traits of 53 cowpea cultivars under NaCl stress by exogenous EBR

**Figure 9:**
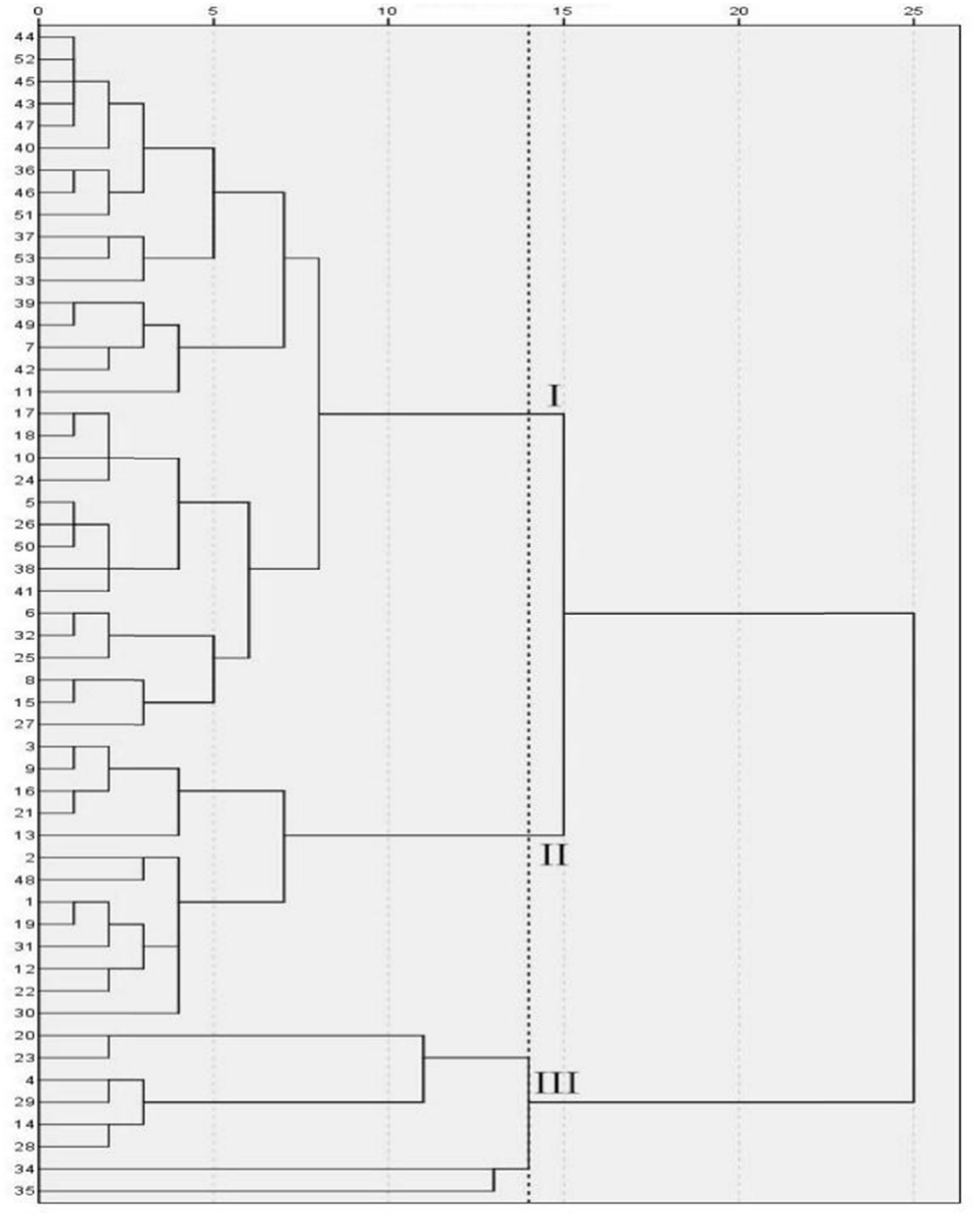
Cluster diagram of cowpea varieties under CK. The vertical axis is the variety number of cowpea, and the horizontal axis is the Euclidean distance. When the genetic distance is 14, the 53 cowpea varieties were clustered into three categories under no treatment.

As shown in Figure 9 and Table 12, when the genetic distance is 14, the 53 cowpea varieties were clustered into three categories under no treatment. Category I consisted of 32 varieties in total, their variety serial number was 5, 6, 7, 8, 10, 11, 15, 17, 18, 24, 25, 26, 27, 32, 33, 36, 37, 38, 39, 40, 41, 42, 43, 44, 45, 46, 47, 49, 50, 51, 52, and 53, respectively. In Category I, Pn, Gs, Ci, and Tr had the maximum values, and Chla, Chlb, Car, Chl(a + b), and Fv/Fm had the minimum values. The variety serial numbers of category II were 1, 2, 3, 9, 12, 13, 16, 19, 21, 22, 30, 31, and 48, consisting of a total of 13 varieties. Pn, Gs, Ci, Tr, Chla, Chlb, Car, Chl(a + b), and Fv/Fm were at medium level. The variety serial numbers of category III were 4, 14, 20, 23, 28, 29, 34, and 35, consisting of a total of eight varieties. Pn, Gs, Ci, and Tr in this category had the minimum values, and Chla, Chlb, Car, Chl (a + b), and Fv/Fm had the largest values.

**Table 12:** Average values of three groups of cowpea varieties under CK

Properties\groups	Ⅰ	Ⅱ	Ⅲ
Pn (CO_2_ μmol m^−2^ s^−1^)	11.804	11.297	8.696
Gs (H_2_O mol m^−2^ s^−1^)	0.620	0.289	0.137
Ci (CO_2_ μmol mol^−1^)	340.985	308.909	276.533
Tr (H_2_O mmol m^−2^ s^−1^)	3.723	2.883	1.697
Rubisco (mg/g)	25.503	22.529	20.885
Chla (mg/g)	0.523	0.597	0.639
Chlb (mg/g)	0.187	0.211	0.228
Car (mg/g)	0.106	0.119	0.129
Chl(a + b) (mg/g)	0.710	0.808	0.866
Leaf length (cm)	7.553	7.395	7.538
Leaf width (cm)	4.775	4.829	4.543
Y(II)	0.597	0.663	0.663
qP	0.888	0.910	0.904
NPQ	0.042	0.050	0.040
Fv/Fm	0.680	0.735	0.741
ARI1	0.015	0.015	0.015
CRI1	0.034	0.037	0.034
PRI	0.024	0.023	0.020
PSRI	0.029	0.025	0.025

**Figure 10:**
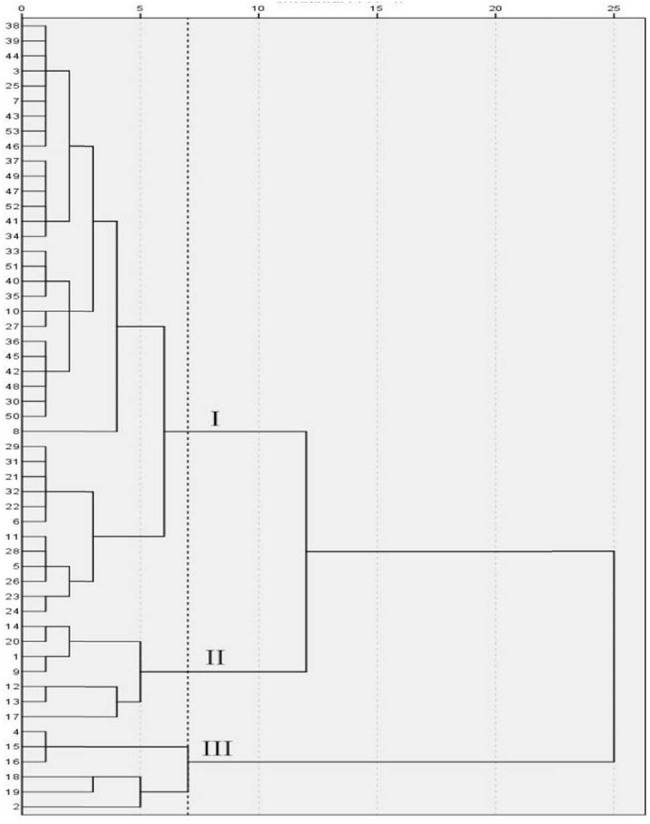
Cluster diagram of cowpea varieties under NaCl stress. The vertical axis is the variety number of cowpea, and the horizontal axis is the Euclidean distance. When the genetic distance was 7, the 53 cowpea varieties were clustered into three categories.

Figure 10 and Table 13 show that under the treatment of salt stress, when the genetic distance was 7, the 53 cowpea varieties were clustered into three categories. The variety serial numbers of category I were 3, 5, 6, 7, 8, 10, 11, 21, 22, 23, 24, 25, 26, 27, 28, 29, 30, 31, 32, 33, 34, 35, 36, 37, 38, 39, 40, 41, 42, 43, 44, 45, 46, 47, 49, 50, 51, 52, 53, consisting of a total of 40 varieties. Pn, Gs, Ci, Tr, Chla, Chlb, Car, Chl(a + b), and ARI1 were at medium levels. The variety serial numbers of the second major category were 1, 9, 12, 13, 14, 17, and 20, respectively, composed of a total of seven varieties. Pn, Gs, Ci, and Tr of this category had the maximum values, and Chla, Chlb, Car and Chl(a + b) had the minimum values. The variety serial numbers of the third category were 2, 4, 15, 16, 18, and 19, composed of a total of six varieties. Pn, Gs, Ci, Tr, Chla, Chlb, Car, Chl (a + b), and ARI1 of this category exhibited the maximum values.

**Table 13:** Average values of three groups of cowpea varieties under NaCl stress

Properties\groups	Ⅰ	Ⅱ	Ⅲ
Pn (CO_2_ μmol m^−2^ s^−1^)	0.8349	0.6694	0.9252
Gs (H_2_O mol m^−2^ s^−1^)	0.0226	0.0069	0.0030
Ci (CO_2_ μmol mol^−1^)	349.0479	534.5957	880.5083
Tr (H_2_O mmol m^−2^ s^−1^)	0.2765	0.0931	0.0563
Rubisco (mg/g)	20.209	16.0734	16.6605
Chla (mg/g)	0.4673	0.4309	0.482
Chlb (mg/g)	0.1687	0.1564	0.1877
Car (mg/g)	0.1056	0.1001	0.1095
Chl(a + b) (mg/g)	0.6359	0.5873	0.6695
Leaf length (cm)	7.0818	6.8881	6.9112
Leaf width (cm)	4.6668	4.3761	4.3833
Y(II)	0.6937	0.6941	0.6577
qP	0.9245	0.9215	0.8775
NPQ	0.0472	0.0332	0.0439
Fv/Fm	0.7600	0.7599	0.7605
ARI1	0.0149	0.0149	0.0159
CRI1	0.0322	0.0329	0.0363
PRI	0.0289	0.0297	0.0254
PSRI	0.0304	0.0320	0.0274

**Figure 11:**
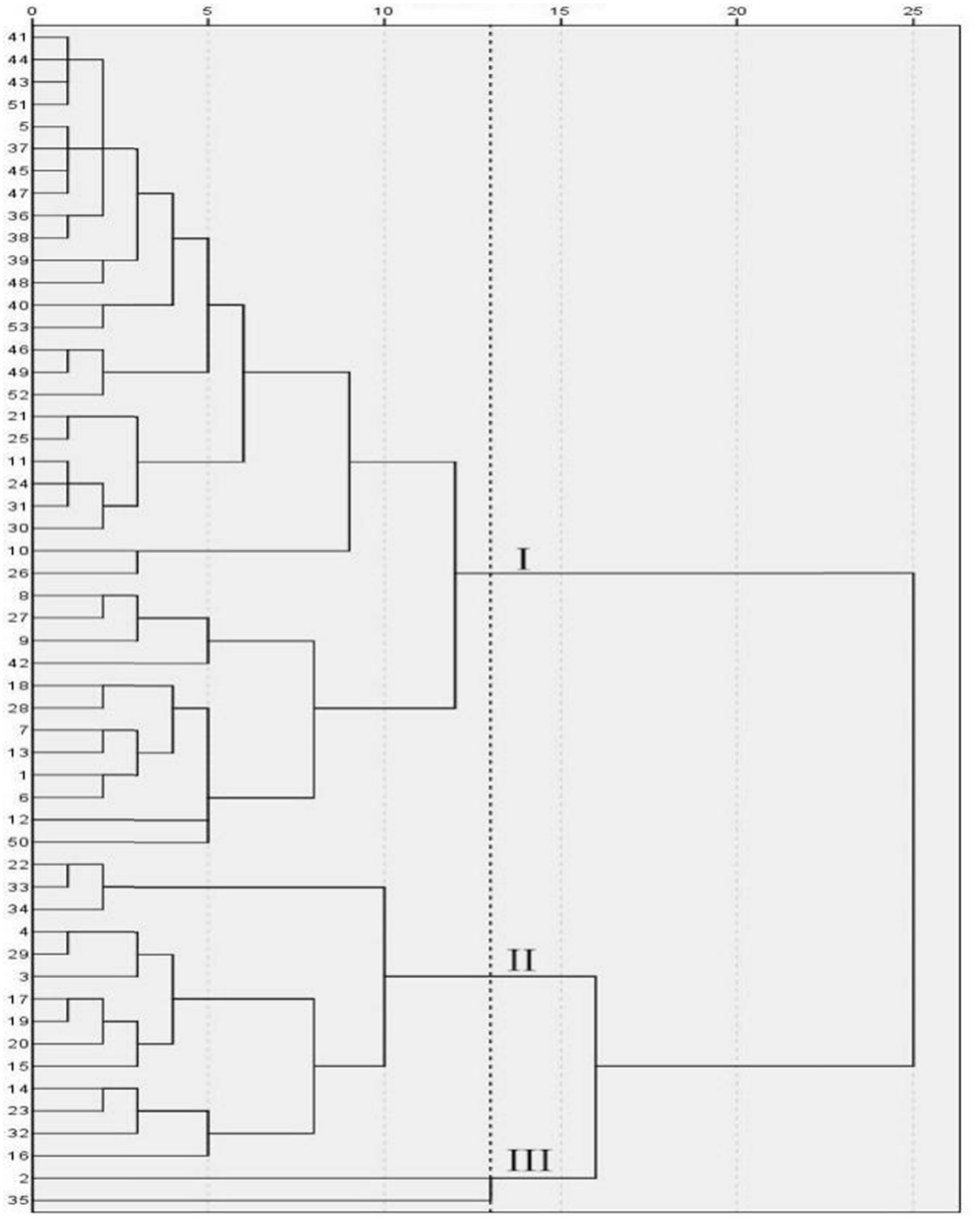
Cluster diagram of cowpea varieties under EBR treatment. The vertical axis is the variety number of cowpea, and the horizontal axis is the Euclidean distance. When the genetic distance was 13, 53 cowpea varieties were clustered into three categories.

It can be seen from Figure 11 and Table 14 that under the treatment of EBR spraying, when the genetic distance was 13, 53 cowpea varieties were clustered into three categories. The variety serial numbers of category I were 1, 5, 6, 7, 8, 9, 10, 11, 12, 13, 18, 21, 24, 25, 26, 27, 28, 30, 31, 34, 36, 37, 38, 39, 40, 41, 42, 43, 44, 45, 46, 47, 49, 50, 51, 52, 53, composed of 37 varieties in total. Pn, Gs, Ci, Tr, and PSRI of this category exhibited the largest values, and Chla, Chlb, Car, Chl(a + b), ARI1, and CRI1 displayed the smallest values. The variety serial numbers of category II were 3, 4, 14, 15, 16, 17, 19, 20, 22, 23, 29, 32, 33, and 34, consisting of a total of 14 varieties. Pn, Gs, Ci, Tr, Chla, Chlb, Car, Chl(a + b), ARI1, CRI1, and PSRI in this category had medium values. The variety serial numbers of category III were 2 and 35, consisting of a total of two varieties. Pn, Gs, Ci, Tr, and PSRI of category III exhibited the minimum values, and Chla, Chlb, Car, Chl(a + b), ARI1, and CRI1 displayed the largest values.

**Table 14:** Average values of three groups of cowpea varieties under EBR treatment

Properties\groups	Ⅰ	Ⅱ	Ⅲ
Pn (CO_2_CO_2_ μmol m^−2^ s^−1^)	11.7143	10.1909	9.0969
Gs (H_2_O mol m^−2^ s^−1^)	0.6444	0.2379	0.1279
Ci (CO_2_ μmol mol^−1^)	343.468	303.9756	270.9729
Tr (H_2_O mmol m^−2^ s^−1^)	3.6915	2.6167	1.7050
Rubisco (mg/g)	29.6558	28.0188	29.4683
Chla (mg/g)	0.5365	0.6151	0.6627
Chlb (mg/g)	0.1895	0.2278	0.2403
Car (mg/g)	0.1100	0.1165	0.1254
Chl(a + b) (mg/g)	0.7258	0.843	0.9027
Leaf length (cm)	7.3022	7.3249	7.1523
Leaf width (cm)	4.7778	4.7501	4.5049
Y(II)	0.6050	0.6426	0.6540
qP	0.8963	0.8957	0.9193
NPQ	0.0529	0.0529	0.0343
Fv/Fm	0.6868	0.7293	0.7184
ARI1	0.0150	0.0155	0.0161
CRI1	0.0334	0.0337	0.0387
PRI	0.0258	0.0262	0.0183
PSRI	0.0281	0.0273	0.0208

**Figure 12:**
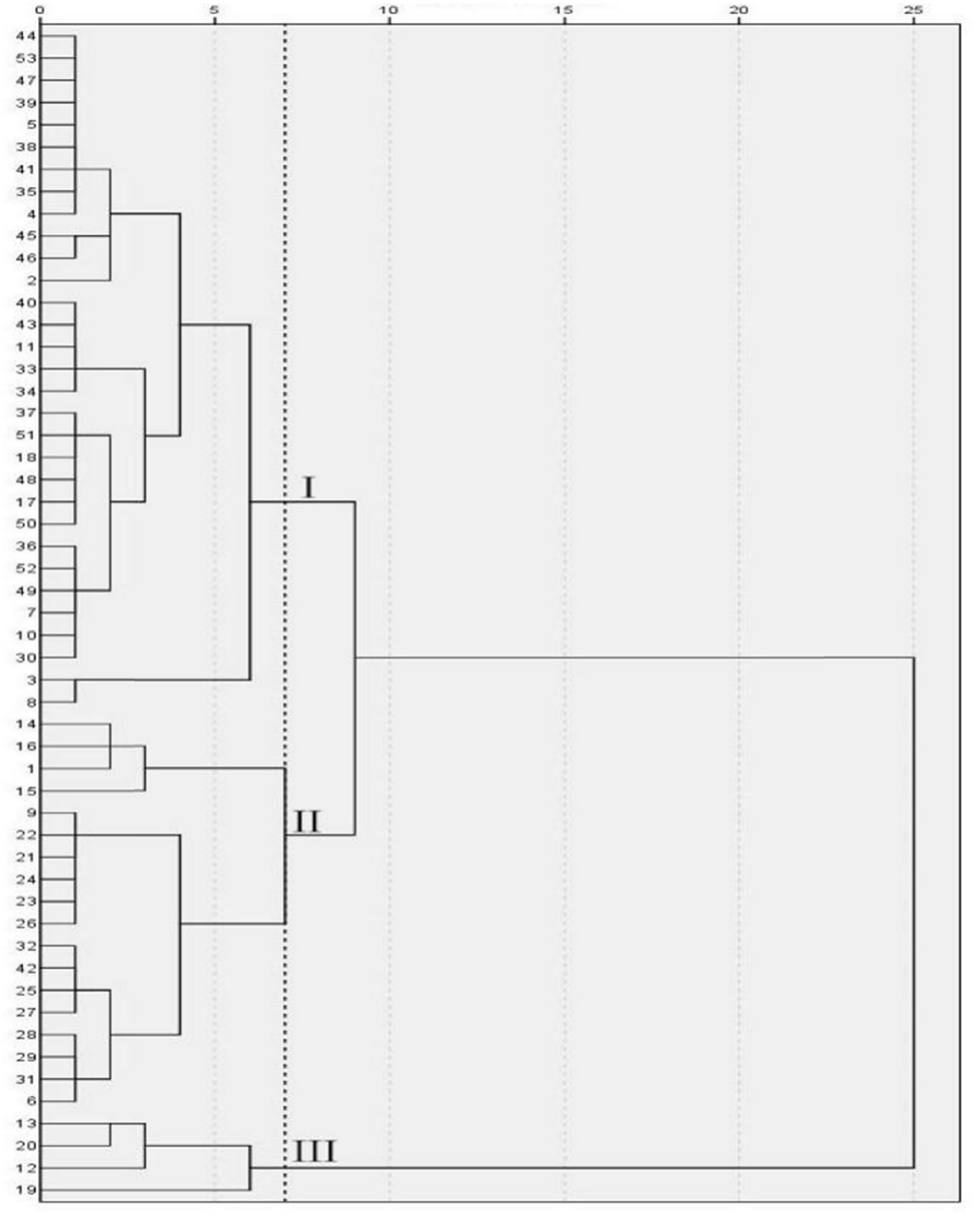
Cluster diagram of cowpea varieties under EBR treatment and NaCl stress. The vertical axis is the variety number of cowpea, and the horizontal axis is the Euclidean distance. When the genetic distance was 7, the 53 cowpea varieties were clustered into three categories.

Figure 12 and Table 15 show that under the treatment of EBR spraying and salt stress, when the genetic distance was 7, 53 cowpea varieties were clustered into three categories. The variety serial numbers of category I were 2, 3, 4, 5, 7, 8, 10, 11, 17, 18, 30, 33, 34, 35, 36, 37, 38, 39, 40, 41, 43, 44, 45, 46, 47, 48, 49, 50, 51, 52, and 53, consisting of 31 varieties in total. Pn, Ci, Gs, Tr, Chla, Chlb, Car, and Chl(a + b) of category I showed the medium values. The variety serial numbers of category II were 1, 6, 9, 14, 15, 16, 21, 22, 23, 24, 25, 26, 27, 28, 29, 31, 32, and 42, composed of a total of 18 varieties. Pn, Ci, Gs, and Tr of category II exhibited the smallest values, and Chla, Chlb, Car, and Chl(a + b) showed the largest values. The variety serial numbers of category III were 12, 13, 19, and 20, comprised a total of four varieties. Pn, Ci, Gs, and Tr of this category were the largest, and Chla, Chlb, Car, and Chl(a + b) were the smallest.

**Table 15:** Average values of three groups of cowpea varieties under EBR treatment and NaCl stress

Properties\groups	Ⅰ	Ⅱ	Ⅲ
Pn (CO_2_ μmol m^−2^ s^−1^)	0.9055	0.7683	1.0488
Gs (H_2_O mol m^−2^ s^−1^)	0.0196	0.0092	0.0020
Ci (CO_2_ μmol mol^−1^)	326.1435	513.6056	928.1825
Tr (H_2_O mmol m^−2^ s^−1^)	0.2441	0.1197	0.0408
Rubisco (mg/g)	25.2989	17.1284	15.5033
Chla (mg/g)	0.4270	0.4475	0.3920
Chlb (mg/g)	0.1574	0.1638	0.1453
Car (g/g)	0.0984	0.1006	0.0945
Chl(a + b) (mg/g)	0.5843	0.6113	0.5373
Leaf length (cm)	7.3258	7.0722	6.7165
Leaf width (cm)	4.6473	4.5166	4.4335
Y(II)	0.6922	0.7073	0.6788
qP	0.9189	0.9353	0.888
NPQ	0.0344	0.0356	0.0347
Fv/Fm	0.7588	0.7616	0.7720
ARI1	0.0149	0.0146	0.0160
CRI1	0.0340	0.0344	0.0372
PRI	0.0304	0.0272	0.0297
PSRI	0.0254	0.0243	0.0262

## 4 Discussion

Germplasm resources are the basis for breeding new varieties. The diversity of germplasm resources includes the species diversity and genetic diversity, and genetic diversity is the core of biological diversity. The research on genetic diversity of germplasm resources includes four aspects: phenotype, cytology, biochemistry, and molecular level. Phenotypic traits are the external characteristics of plants during growth and development, which are morphologically marked by visual observation or instrumental measurement [13–15]. The results of the different analysis in this study showed that in CK, the largest one of CVs of 19 photosynthetic physiological traits reached up to 76.483% and the smallest one was 7.235%, CVs of 16 out of 19 traits exceeded 10%, indicating that most of the 53 cowpea varieties exhibited significantly different photosynthetic physiological traits, with relatively rich genetic diversity. Under NaCl stress, the largest one of CVs of the 19 photosynthetic physiological traits was 86.645%, and the smallest one was 5.503%, of which CVs of 16 traits exceeded 10%, suggesting that under NaCl stress, most of the photosynthetic physiological traits of these 53 cowpea varieties were significantly different. Through the examination of photosynthetic response, salt tolerance, and other indicators of cowpeas under salt stress, Praxedes et al. [16] found that different cowpea varieties also exhibited different degrees of variability under salt stress, consistent with the results of this article that showed variations in photosynthetic characteristics among different cowpea varieties under NaCl stress. Under the EBR treatment, the largest one of CVs of 19 photosynthetic physiological traits reached up to 103.782%, and the smallest one was 5.805%, and the CVs of 15 out of 19 traits exceeded 10%, implying that the effects of EBR on most of the photosynthetic physiological traits of these 53 cowpea varieties were significantly different. Under the EBR treatment and NaCl stress, the largest one of CVs of 19 photosynthetic physiological traits was up to 71.130%, and the smallest one was 5.745%. Of 19 traits, CVs of 16 traits exceeded 10%, indicating that the alleviation effects of EBR on most photosynthetic physiological traits of these 53 cowpea varieties under NaCl stress were significantly different. Sousa et al. [17] showed that EBR plays a positive role in plants and can, to some extent, alleviate some of the negative impacts of salt stress on cowpeas, such as enzyme activity and photosynthesis capacity.

Somayyeh et al. [10] have reported that after the pods were removed at the pod stage of cowpea, the flower drop rate was negatively correlated with Fv/Fm, PRI, chlorophyll content, and yield, but not significantly. Yield was negatively correlated with Fv/Fm, PRI, and chlorophyll content, but not significantly. Liu et al. [11] have studied the correlation among chlorophyll fluorescence parameters, spectral parameters, photosynthetic parameters, and yield of vegetable soybean at the flowering stage, and found that Pn was extremely significantly positively correlated with PRI, and that leaf Tr was extremely significantly positively correlated with leaf Gs and PSRI. The results of correlation analysis of photosynthetic traits in this study showed that Pn and Tr were extremely significantly positively correlated, and that Pn and Gs were significantly positively correlated in all treatments. Pn and Ci were extremely significantly positively correlated under C and E treatments in the control group, whereas they were negatively correlated under N and EN treatments, but not significantly. Pn was significantly negatively correlated with Chla, Chlb, and Chl(a + b) under C and E treatments in the control group, whereas they were negatively correlated under N and EN treatments but not significantly.

PCA is a method of condensing information through the dimensionality reduction. After the data transformation, the newly generated variables represent most of the original information, and they can be used for PCA. PCA-based germplasm resource evaluation has been conducted in grape [18], tomato [19], corn [20], wheat [21], lentils [22], cowpea [23,24], and other crops successively. The results of PCA in this study showed that in CK, 19 photosynthetic physiological traits of 53 cowpea varieties were assigned into six principal components with a cumulative contribution rate of 82.298%, reflecting 82.298% of the total amount of information. Under NaCl stress, the 19 photosynthetic physiological traits of 53 cowpea varieties were assigned into seven principal components with a cumulative contribution rate of 84.564%, reflecting 84.564% of the total amount of information. Under EBR treatment, 19 physiological traits were assigned into six principal components with a cumulative contribution rate of 83.046%, reflecting 83.046% of the total amount of information. Under EBR treatment and NaCl stress, 19 photosynthetic physiological traits of 53 cowpea varieties were assigned into seven principal components, with a cumulative contribution rate of 85.742%, reflecting 85.742% of the total amount of information.

Cluster analysis can reflect the genetic differences and genetic relationship among different varieties, and it can provide a certain reference for formulating breeding schemes. Cluster analysis has been used to select target traits accordingly, in chrysanthemum [25], pea [26], cowpea [27], strawberry [28], barley [29], and many other crops. Our cluster analysis results showed that without treatment, the 53 cowpea varieties were clustered into three categories. The category I of cowpea varieties exhibited the strongest photosynthetic ability, which can be used to breed cowpea varieties with high light efficiency. The photosynthetic ability of category II cowpea varieties was at medium level, but its comprehensive ability was better. The photosynthetic ability of category III cowpea varieties was the worst, and thus it should not be recommended being used for breeding high light-efficiency cowpea varieties. Under the treatment of salt stress, the 53 cowpea varieties were clustered into three categories. The photosynthetic ability of category I under salt stress was average, which belonged to moderate salt-tolerant cowpea varieties. The photosynthetic ability of category II was the weakest, which belonged to the salt-sensitive cowpea variety. The photosynthetic ability of category III was the strongest under salt stress, and belonged to the salt-tolerant cowpea variety. Under the treatment of EBR spraying, 53 cowpea varieties can be grouped into three categories. Under the treatment of EBR, the photosynthetic ability of category I was the strongest, which was suitable for EBR fertilization. The photosynthetic ability of category II was average, and the comprehensive ability was better. Under the treatment of EBR, the photosynthetic ability of category Ⅲ cowpea varieties was the worst, which was not suitable for EBR fertilization. Under EBR spraying + salt stress, the 53 cowpea varieties were clustered into three categories. EBR had a moderate mitigation effect on the photosynthesis of category I cowpea varieties under salt stress. EBR exhibited the worst mitigation effect on the photosynthesis of category II cowpea varieties under salt stress, and thus EBR was not suitable to alleviate the salt damage to this category of cowpea varieties. EBR had the strongest mitigation effect on the photosynthesis of category III cowpea variety under salt stress, and thus it was suitable for EBR to mitigate salt damage to this category cowpea variety.

## 5 Conclusion

In this study, we conducted difference, correlation, principal component, and cluster analyses to investigate the photosynthetic traits of 53 cowpea germplasm resources under salt stress after application of EBR. The results of different analysis and correlation analyses showed that these 53 germplasm resources had rich genetic diversity, and there were correlations among their different agronomic traits to various degrees. The 32 varieties (with variety serial numbers as 5, 6, 7, 8, 10, 11, 15, 17, 18, 24, 25, 26, 27, 32, 33, 36, 37, 38, 39, 40, 41, 42, 43, 44, 45, 46, 47, 49, 50, 51, 52, and 53) were screened by combining PCA and the cluster analysis. They had the strongest photosynthetic ability, and could be used to breed cowpea varieties with high light efficiency. Under the treatment of salt stress, six varieties (2, 4, 15, 16, 18, and 19) belonged to the salt-tolerant cowpea varieties. Under EBR treatment, 31 varieties (2, 3, 4, 5, 7, 8, 10, 11, 17, 18, 30, 33, 34, 35, 36, 37, 38, 39, 40, 41, 43, 44, 45, 46, 47, 48, 49, 50, 51, 52, and 53) exhibited the strongest photosynthetic ability, and thus they were suitable for EBR fertilization. Under the treatment of EBR spraying + salt stress, the four varieties (12, 13, 19, and 20) displayed excellent photosynthetic properties, and the strongest mitigation effect of EBR. Taken together, this study revealed the differences in the physiological responses of different cowpea varieties to exogenous EBR. Our findings will provide a reference for the application of exogenous EBR to alleviate salt stress damage to cowpea.
